# Blood donor deferrals: Can this be reduced?

**DOI:** 10.4103/0973-6247.76011

**Published:** 2011-01

**Authors:** Kusum D. Jashnani, Laxmi N. Patil

**Affiliations:** Department of Pathology, T.N. Medical College & BYL Nair Ch Hospital, Mumbai Central, Mumbai 400 008, India

Sir,

The information regarding selection criteria for blood donation if made available to prospective blood donors prior to the day of donation may help reduce deferrals. One should also make an effort to avoid any psychological effect or negative impact on the prospective donors, which may prevent them from future donations. Against 1500 donors accepted for donation, the total number of donors deferred was studied. In prospective study, the deferred donors were interviewed personally to find out cause of rejection, their level of education, impact on donor, and willingness to donate in future (in case of temporary deferrals). The number of deferrals against 800 blood donations was evaluated. In retrospective study, the data about donor deferrals was collected from the donor deferral registry of the blood bank, though the information on impact on the donor, education level, and willingness to donate was not available. The number of donors deferred against a total of 700 donors accepted for donation was evaluated. A total of 378 (25.2%) donors were deferred against 1500 accepted donors. Of these total deferred donors, 49.5% belonged to long-term category (61-730 days), while 43.7% belonged to short-term category (1-60 days). Permanent deferrals constituted 6.8% of total deferrals.

Low hemoglobin (27.5%) was commonest cause of deferral in female donors. It has been reported that 95% of deferrals for low hemoglobin occur in women. It has also been suggested that hemoglobin standard be lowered to increase female eligibility and to offer iron treatment for premenopausal woman who want to donate or who are frequent donors.[1,2] The second most common cause of predonation deferral was high BP (12.4%) especially in males. High BP in some was diagnosed for first time, while rest were the cases of uncontrolled hypertension on medication. Prospective donors on regular antihypertensive therapy can get their BP checked by their family physician few days prior to the donation camp if they are aware of uncontrolled BP being a cause of temporary deferral. Intake of medication (8.7%) by donors was third cause. This included analgesics and antibiotics for which donor is deferred for few days, to drugs like anti-Koch’s treatment or ayurvedic medicines for which donor is deferred for 1-2 years. Alcohol intake within last 24 hours (7.1%), the fourth common cause, is easily avoidable. If a practice is made by the concerned blood bank to educate the main organizer or the organizing team of the blood camp as regards to some of the causes of the temporary deferrals, few days prior to the date of donation camp, we are sure the number of deferrals would definitely reduce. Suffering from cold, cough, and fever at the time of donation was a cause of deferral in 4.4% cases. This may be simple viral infection, which requires deferral of few days, or the cough may be of long duration and suggestive of tuberculosis (which is common in our country) requiring the donor to take medical advice, in which cases the deferral is for few years. Low BP was another important cause observed in the study. It would be the fear of donation and the sight of blood. Unwillingness of donor to donate (1.1%) in spite of being healthy and screened positively through all tests and examination was observed. This was due to fear of contracting blood-transmitted diseases (especially HIV) due to needle prick and unsatisfied with arrangements made. No convenient place to donate and poor staff skills was a common cited reason for not returning for donation.[3]

History of sexual exposure was observed as a commonest cause of permanent deferral especially in the younger age group between 18 to 30 years. It, being a permanent cause of deferral, can lead to tremendous loss in future blood donation; 189 deferred donors were disappointed and sad as against 5, 2, and 2 were tensed, surprised about their health status, and angry with the blood bank staff, respectively. Level of education did not have any influence on the percentage of deferrals. There were equal number of graduates and less-educated prospective donors who were deferred. Similar finding was observed in Turkish population.[4] Most of temporary deferrals (86.6%) in prospective study agreed to return for donation in future. It is important to provide donors with a clear message on their deferral status. Deferrals for whatever reason represent loss of time and effort for both potential donors and blood bank staff.
